# Fragment C of Tetanus Toxin: New Insights into Its Neuronal Signaling Pathway

**DOI:** 10.3390/ijms13066883

**Published:** 2012-06-07

**Authors:** Ana C. Calvo, Sara Oliván, Raquel Manzano, Pilar Zaragoza, José Aguilera, Rosario Osta

**Affiliations:** 1 LAGENBIO (Laboratory of Genetics and Biochemistry), Faculty of Veterinary-I3A, Aragonese Institute of Health Sciences (IACS), University of Zaragoza, Miguel Servet 177, 50013 Zaragoza, Spain; E-Mails: accalvo@unizar.es (A.C.C.); soligar@unizar.es (S.O.); rmanzano@unizar.es (R.M.); pilarzar@unizar.es, osta@unizar.es (P.Z.); 2 Institute of Neurosciences, Department of Biochemistry and Molecular Biology, Universitat Autònoma de Barcelona (UAB), Center of Biomedical Research Network in Neurodegenerative Diseases (CIBERNET), 08193, Cerdanyola del Vallès, Spain; E-Mail: jose.aguilera@uab.cat

**Keywords:** clathrin-mediated pathway, dynamin, fragment C, tetanus toxin, neurotrophin, Trk receptors

## Abstract

When *Clostridium tetani* was discovered and identified as a Gram-positive anaerobic bacterium of the genus *Clostridium*, the possibility of turning its toxin into a valuable biological carrier to ameliorate neurodegenerative processes was inconceivable. However, the non-toxic carboxy-terminal fragment of the tetanus toxin heavy chain (fragment C) can be retrogradely transported to the central nervous system; therefore, fragment C has been used as a valuable biological carrier of neurotrophic factors to ameliorate neurodegenerative processes. More recently, the neuroprotective properties of fragment C have also been described *in vitro* and *in vivo*, involving the activation of Akt kinase and extracellular signal-regulated kinase (ERK) signaling cascades through neurotrophin tyrosine kinase (Trk) receptors. Although the precise mechanism of the molecular internalization of fragment C in neuronal cells remains unknown, fragment C could be internalized and translocated into the neuronal cytosol through a clathrin-mediated pathway dependent on proteins, such as dynamin and AP-2. In this review, the origins, molecular properties and possible signaling pathways of fragment C are reviewed to understand the biochemical characteristics of its intracellular and synaptic transport.

## 1. Introduction

The *Clostridial* neurotoxin family comprises tetanus neurotoxin and seven distinct botulinum neurotoxins, which cause the diseases tetanus and botulism [1,2]. Regarding neuroparalytic clostridia, *Clostridial botulinum* and *Clostridial tetani* produce the most potent toxins, botulinum and tetanus, due to their remarkable neurospecificity and their catalytic cleavage at low concentrations of neuronal substrates, which can oscillate between 10^−12^ and 10^−13^ M. The main difference between these toxins is in the intensity and duration of muscle paralysis. In fact, severe tetanus is characterized by violent and persistent spasms of the head, trunk and limb muscles [2].

The infectious nature of tetanus toxin was well documented before 1906, and the necessity of producing a tetanus antitoxin was decisive during the First World War [3]. The promising protective effects of eosin, tested *in vitro* and *in vivo* [4,5], and the development of serums against the toxin, mainly obtained in horses [6], undoubtedly opened a door to the unexplored field, at that time, of its molecular mechanism of action. In 1905, Sherrington described the mechanism of action of the toxin on spinal reflexes as a conversion of inhibition into excitation, thus destroying coordination [7]. Additionally, he investigated the effects of strychnine because this substance had a similar effect on the central nervous system as the tetanus toxin. In 1942, Acheson and collaborators concluded for the first time that the toxin was carried to the spinal cord through peripheral nerves when it was injected intramuscularly in cats; that is, the toxin traveled selectively through the segments of the spinal cord that supplied the innervation of the injected area [8]. Supporting this result, Brooks and collaborators, 15 years later, studied the spinal inhibitory mechanisms based on five types of inhibition [9]:

- direct inhibition of motoneurons by impulses in Group I a afferent fibers of antagonistic muscles;
- inhibition by impulses in the Group I b afferent fibers from muscles of the same limb;
- inhibition of extensor motoneurons by impulses in Groups II and III muscle afferent fibers and in cutaneous afferent fibers; and
- inhibition of motoneurons following the activation of Renshaw cells by volleys in axon collaterals.

These five forms of spinal inhibition were diminished and eventually abolished 8–10 h after injecting the toxin either peripherally into a mixed nerve trunk or directly into the spinal cord, which indicated that the toxin diffused slowly across the spinal cord, taking several hours to move 1 mm. From these results, it was also concluded that the toxin moved much faster longitudinally in nerve trunks and within the spinal cord [9].

The nature of the action of tetanus toxin has been widely described in different animal models [10–15], exploring its effect not only in the spinal cord but also in the cerebral cortex [16]. Different pharmacological substances have been used to ameliorate its symptoms in addition to eosin, such as acetylcholine and eserine [12]. These data were the starting point to attempt to characterize a protein that was completely necessary for the toxin to exert its effect, the tetanus-toxin receptor. Wassermann and Takaki, in 1898, observed that the toxin was fixed in the nervous tissue; in particular, brain tissue was more effective than the spinal cord, and gray matter was more effective than white matter [17]. Eight years later, Landsteiner and Botteri believed that phrenosine was the receptor of the tetanus toxin [18]. However, in 1959 van Heyningen reinvestigated phrenosine as a possible receptor of the toxin, and he realized that the phrenosine concentrations in gray and white matter were inversely proportional to their capacity to fix the toxin. Based on his experiments in bovine brain samples, he concluded that the receptor was a highly specific substance that was mainly present in the gray matter, its interaction with the toxin was unaffected by temperature and it tended to associate with cerebrosides, although it was distinct from them [19,20]. Two years later, he confirmed that the sialic acid residues of the gangliosides were essential for toxin fixation and that the toxin did not appear to change the ganglioside molecule [21]. Although further studies supported this fact [22,23], currently, the identity of this receptor remains unknown despite knowledge of its agonist, tetanus toxin.

## 2. Molecular Structure and Properties of Fragment C: Toward the Discovery of Fragment C

**Figure 1:**
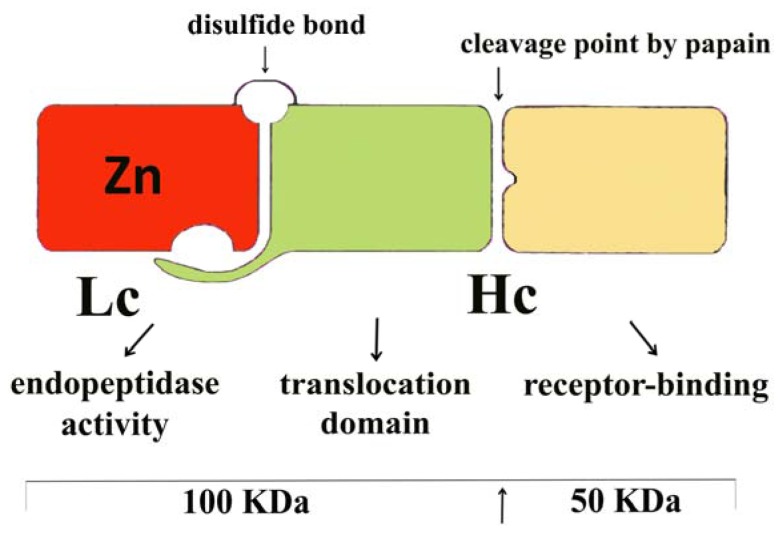
Diagram of the tetanus toxin molecule. The targeting and the translocation domains are located in the heavy-chain (HC), whereas the catalytic domain is located in the light-chain (LC) of the molecule. Its proteolytic activity is Zn^2+^-dependent, and heavy-metal chelators generate inactive apo-neurotoxins. The position of the cleavage of the tetanus-toxin molecule by papain is indicated. The digestion yields two fragments; one of them, fragment C, is approximately 50 kDa [29].

Tetanus toxin is a single peptide of approximately 150 kDa, which consists of 1315 amino-acid residues. The toxin forms a two-chain activated molecule composed of a heavy chain (HC) and a light chain (LC) linked by a disulfide bond. The catalytic domain of the toxin resides in the LC, while the translocation and receptor-binding domains are present in HC [24–27] (Figure 1). Tetanus and botulinum toxins are zinc metalloproteases that cleave SNARE (soluble NSF attachment receptor) proteins, which interfere with the fusion of synaptic vesicles to the plasma membrane and ultimately blocks neurotransmitter release in nerve cells [28].

**Figure 2:**
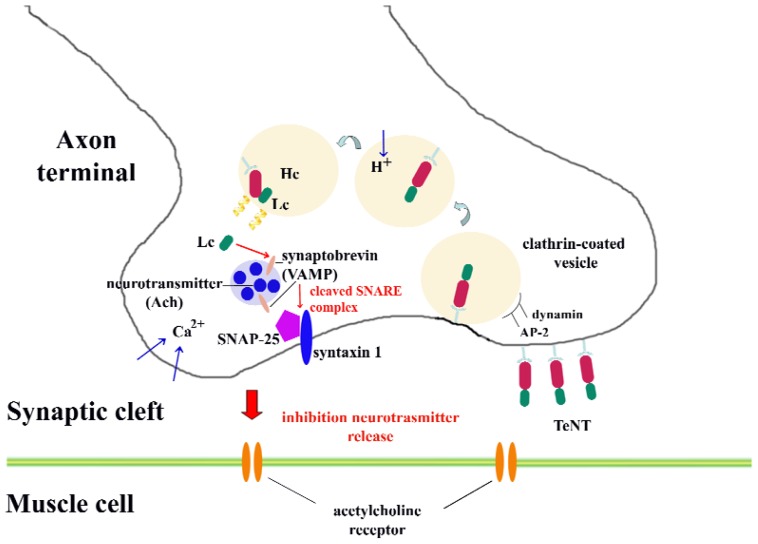
Proposed internalization pathway of tetanus toxin. The ganglioside-recognition domain in the *C*-terminal region of HC allows the toxin to be internalized into the neuron. The light chain of the toxin (LC) cleaves the soluble NSF attachment receptor (SNARE) complex, inhibiting neurotransmitter release.

The mechanism of cell internalization and catalytic activity of tetanus toxin can be summarized in few steps [24,25] (Figure 2):

- cell binding, mainly mediated by the ganglioside-recognition domain in the *C*-terminal region of H_C_;
- internalization into neuronal cells. Different internalization mechanisms have been described: the mechanism that tetanus toxin possibly follows to internalize into neurons may be a clathrin-mediated pathway, which is dependent on the proteins dynamin, AP-2 and AP180 [30]. Other potential receptor molecules will be discussed in the next section.
- membrane translocation from mature endosomes into the neuronal cytoplasm. After incorporation of the toxin within endosomes, a structural change of the toxin is induced by the acidification of the endosomal environment; thus, a membrane-spanning pore forms. At this step, the oligomerization of four toxin amphipathic alpha-helices is required for channel formation; and
- target recognition and catalytic cleavage of neuronal substrates. Once the toxin reaches the cytoplasm, it specifically cleaves neuronal proteins integral to vesicular trafficking and neurotransmitter release. In particular, the synaptic vesicle protein synaptobrevin (VAMP) is the target of tetanus toxin. This protein belongs to a family of proteins that facilitate exocytosis in neurons known as SNARE proteins. The other members of this family are syntaxin and SNAP-25, which are the main molecular targets of botulinum toxin. SNARE proteins are formed by coiled-coil interactions of the alpha-helices of its members, which is required for membrane fusion [31–33].

Botulinum toxins are produced by the anaerobic bacterium *Clostridium botulinum*, which is considered to be a potent blocker of synaptic transmission in peripheral cholinergic nervous system synapses. There are seven serologically distinct botulinum isoforms (denoted A–G), which exhibit strong amino-acid sequence similarity. The mature toxin is characterized by three main structural domains, an *N*-terminal light chain Zn^2+^-metalloprotease and the heavy chain that contains the *N*-terminal, of approximately 50-kDa, translocation domain, and the *C*-terminal, of approximately 50-kDa, receptor-binding domain. This receptor-binding domain comprises two subdomains, a β-sheet jelly-roll fold and a β-tree foil-fold carboxy subdomain. The seven botulinum toxin serotypes cleave specific residues on one of three SNARE proteins: botulinum serotypes B, D, F and G cleave VAMP, botulinum serotypes A and E cleave SNAP25, and botulinum serotype C cleaves SNAP25 and syntaxin 1a [26,28].

### 2.1. Molecular Properties of Fragment C

#### 2.1.1. Molecular Binding

As previously mentioned, tetanus toxin can form channels in lipid membranes at low pH in the endosomal compartment of nerve cells. These channels are actually pores, which are involved in the transport of the toxin to the cytosol [34]. The ability to form pores in lipid vesicles resides in the hydrophobic domain of LC and the *N*-terminal of HC, which allows for the translocation of the toxin through the lipid membrane. However, in the absence of fragment C, the toxin retains little ability to paralyze neuromuscular transmission [35]. The lipids which are most sensitive to the action of the toxin are, in descending order: phosphatidylinositol > phosphatidylserine > phosphatidylcholine and cholesterol [36]. Furthermore, tetanus toxin increases its binding and insertion into lipid bilayer at acidic pH, suggesting that the toxin can penetrate into cells through a low pH intracellular compartment [37].

What is the molecular structure of fragment C that makes it essential for toxin behavior?

The early step in tetanus-toxin internalization is cell binding. Lipid rafts are microdomains of the plasma membrane enriched in sphingolipids (gangliosides are members of this group of lipids), cholesterol and glycosylphosphatidylinositol (GPI)-anchored proteins. Lipid rafts behave as specialized domains for tetanus-toxin binding and internalization into neurons. In particular, gangliosides constitute the main part of mammalian plasma membranes. Their oligosaccharide residues and their ubiquity at the outer leaflet of the membrane allow them to function as bioactive signal transducers. The affinity of fragment C for gangliosides, which resides in the last 34 residues, has been widely tested in different animal models and in different tissues. For example, greater interaction was observed with long-chain gangliosides from rat brain than with similar gangliosides in rabbit kidney [38]. Furthermore, it is suggested that tetanus toxin, mainly through fragment C, and gangliosides undergo significant conformational changes upon their interaction, leading to the formation of macromolecular aggregates. In particular, cerebrosides, sulfatides, sphingomyelin and phosphatidylserine seem to increase the percentage of α-helices in the toxin [39].

**Figure 3:**
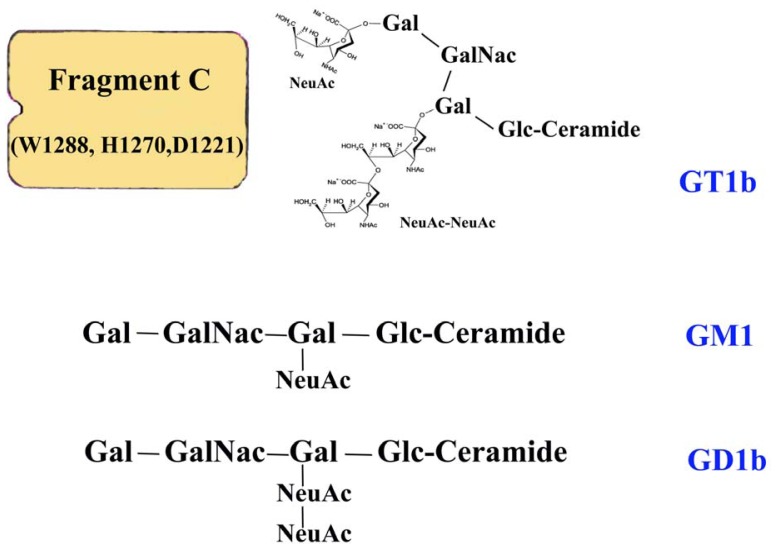
A schematic representation of the structures of gangliosides GT1b, GM1 and GD1b. The strongest and most specific ganglioside association with fragment C of tetanus toxin is with GT1b, since the targeting domain of the toxin contains two binding sites that can accommodate NeuAc residues.

Interestingly, studies on the ganglioside-binding properties of fragment C have demonstrated that polysialic acids within the gangliosides, such as GD1b (disialic acid residues attached to the internal galactose residue) and GT1b (disialic acid residues attached to the terminal galactose residue), but not GM1 (monosialic acid residue attached to the internal galactose residue), are necessary for the binding process [40,41]. Specifically, the amino-acid residues tryptophan 1288, histidine 1270 and aspartate 1221 were found to be critical for the binding of fragment C to ganglioside GT1b [42]. GT1b is a trisialo sphingolipid with a branched carbohydrate structure containing a single *N*-acetylneuraminic acid (NeuAc) on one arm and a NeuAc dimer on the other (Figure 3). The strongest and most specific ganglioside association with tetanus toxin is with GT1b because the targeting domain of the toxin contains two binding sites that can accommodate NeuAc residues separated by a distance of approximately 25 Å [43]. Studies based on atomic-force microscopy/total internal-reflection fluorescence microscopy (TMAFM/TIRFM) reinforce this result, suggesting that the membrane activity of fragment C is dependent on both the ganglioside concentration in the membrane and the pH of the medium [44].

**Figure 4:**
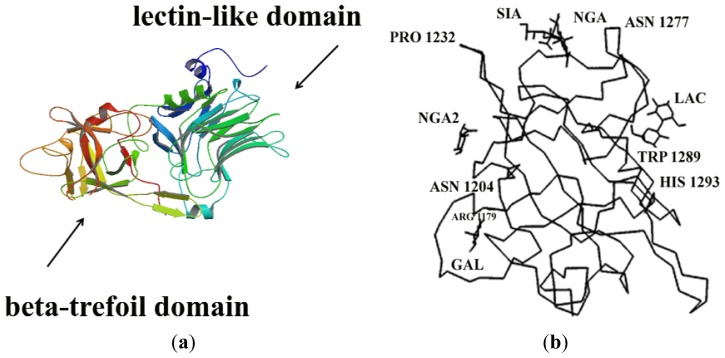
(**a**) Image from the RCSB PD of PDB ID 1A8D [47]; (**b**) Illustration of the carbohydrate-binding sites for lactose (LAC), galactose (GAL), sialic acid (SIA) and *N*-acetyl-galactosamine (NGA) in tetanus toxin. These binding sites are localized in the beta-trefoil domain of fragment C (residues 865–1315). Adapted from [40].

Additionally, the receptor-binding domain of tetanus and botulinum A and B toxins is structurally similar, containing two subdomains: an amino-terminal lectin-like jelly-roll subdomain and a carboxyl-terminal beta-trefoil subdomain linked by a single chain (Figure 4). Each of these subdomains is composed of beta-sheets joined by loops that protrude from the molecule. Regarding tetanus toxin, and based on previous studies [19–21,45], four distinct carbohydrate-binding sites for lactose, galactose, sialic acid and N-acetyl-galactosamine (NGA) were determined with X-ray crystallography [40,41]. In particular, the beta-trefoil subdomain seems to have a more relevant role in ganglioside binding than does the amino-terminal lectin-like subdomain, which was demonstrated by analyzing the localization of these binding domains, showing that the binding function resided in the beta-trefoil domain [40,46] (Figure 4), and mutant proteins of fragment C [41].

**Figure 5:**
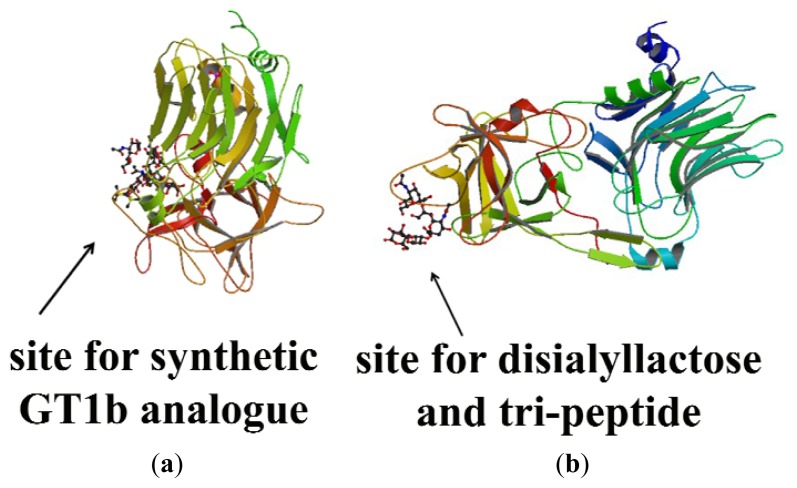
(**a**) Image from the RCSB PDB of PDB ID 1FV3 regarding Site-1 of fragment C [51]; (**b**) Image from the RCSB PDB of PDB ID 1YYN regarding Site-2 of fragment C [52].

Based on studies that included a combination of computational methods, these four carbohydrate-binding sites are localized in two different sites on the surface of fragment C: Site-1, a common site for all clostridial neurotoxins through which fragment C binds lactose and part of GT1b (Gal-GalNAc); and the other well-characterized site Site-2, through which fragment C binds sialyllactose, lactose, disialyllactose (DiSia), a tripeptide Tyr-Glu-Trp (YEW) and the Gal-NAc part of GT1b. Site-2 has been shown to be vital for the toxicity of the toxin [48]. Furthermore, Tyr 1290 and Trp 1289 in Site-1 and Asp 1147 and Arg 1226 in Site-2 play a key role in ganglioside binding [49] (Figure 5).

In particular, DiSia binding characterizes ganglioside binding to fragment C and could be the binding site for the ganglioside sugar moiety GD3 [48,50]. If ligands that simultaneously bind to two adjacent sites are identified, they could be used to develop bi-dentate reagents for tetanus toxin, as demonstrated by Cosman and collaborators. Of the six small molecules they studied, three were found to be the best ligands to use for preparing bi-dentate detection agents: doxorubicin or 3′-sialyllactose (binding to Site-1), lavendustin A, YEW and MP-biocytin (binding to Site-2) and the peptide Ser-Gln-Asn-Tyr-Pro-Ile-Val (SQNYPIV) (binding to a third independent site). Doxorubicin presents more advantages than do the other molecules because it tends to bind fragment C over a wider range of temperatures, solvent conditions and concentrations [50].

More recent studies have suggested that gangliosides are functional dual receptors for tetanus toxin and they are necessary for high-affinity binding to neuronal and non-neuronal cells. In particular, these studies showed that HC of the toxin bound gangliosides via two carbohydrate-binding sites, the lactose binding site called W-pocket and the sialic acid binding site called R-pocket. Both W- and R-pockets are the binding sites for GM1a and GD3 gangliosides [53].

Although the affinity of fragment C for gangliosides has been widely characterized, another hypothesis could suggest that a high-affinity protein receptor can be involved in tetanus toxin internalization. Schiavo and co-workers showed that the *N*-glycosylated 15-kD receptor protein has also been described as a surface glycoprotein that interacts with tetanus toxin in neuronal cell-lines and motor neurons. The *C*-terminal subdomain of fragment C of tetanus toxin is sufficient and necessary for cell binding and interaction with the 15-kD putative receptor, highlighting the importance of this domain of fragment C for the neurospecific interaction of the toxin [54]. The same group also suggested that GPI anchored protein Thy-1, a highly expressed glycoprotein that can interact with tetanus toxin to mimic ganglioside binding [55]. In addition, HC of the toxin can be retrograde-trafficked within motor neurons in Rab-7 positive structures that are shared with the neurotrophin receptors p75^NRT^ and TrkB [56]. Swaminathan and co-workers showed that a tri-peptide Tyr-Glu-Trp bound to the binding-receptor domain of the toxin with interactions to Arg^1226^, suggesting that a protein as well as a ganglioside can bind to this domain [49].

Similarly to tetanus toxin, botulinum toxin Types A, B, C and F bind gangliosides GT1b, GD1b and GD1a, whereas botulinum Type E binds GT1b and GT1a, botulinum Type D binds phosphatidylethanolamine (PE) and botulinum Type G recognizes all the gangliosides with an approximately similar affinity [26]. Furthermore, the described surface-protein receptors, involved in neuronal specificity of botulinum toxins, are related to protein components of the synaptic vesicle membrane. Interestingly, the botulinum serotypes that exhibit highest sequence similarity share the same protein receptor. This is the case of botulinum Types A, E and F, which bind SV2, a family of synaptic-vesicle membrane proteins, whereas botulinum Types B, and G bind SytI and SytII, which is the calcium sensor that triggers synaptic vesicle fusion. No protein receptor has been associated with botulinum Types C and D. The general pathway of nerve entry begins with the preinsertion conformation of the translocation domain, followed by the translocation of the light-chain protease across endosomes. This translocation is highly dependent on the pH gradient, redox gradient and the transmembrane potential. The completion of translocation ends when the *C*-terminus of the light chain enters the cytosol, where SNARE cleveage takes place and this is the last portion translocated that exit the channel. The disulfide bridge between light and heavy chains is crucial for botulinum toxicity and is required for chaperone function, acting as a principal determinant for cargo translocation and release [26].

#### 2.1.2. Retrograde Axonal Transport

One of the unique characteristics of tetanus toxin is that it can be transported retrogradely to the central nervous system from the circulatory system. The first question was whether fragment C itself could be transported by neurons similar to the native toxin while avoiding its toxicity. The trans-synaptic transport of fragment C was intensively studied in one of the best-characterized systems, the primary visual pathway [57,58], confirming its capacity as a carrier once it was injected intramuscularly [59]. Furthermore, the possibility of constructing hybrid molecules with fragment C has opened the door to an interesting research field, the discovery of neuro-anatomical tracers, whose main purpose is to map synaptic connections between neuronal cells.

**Figure 6:**
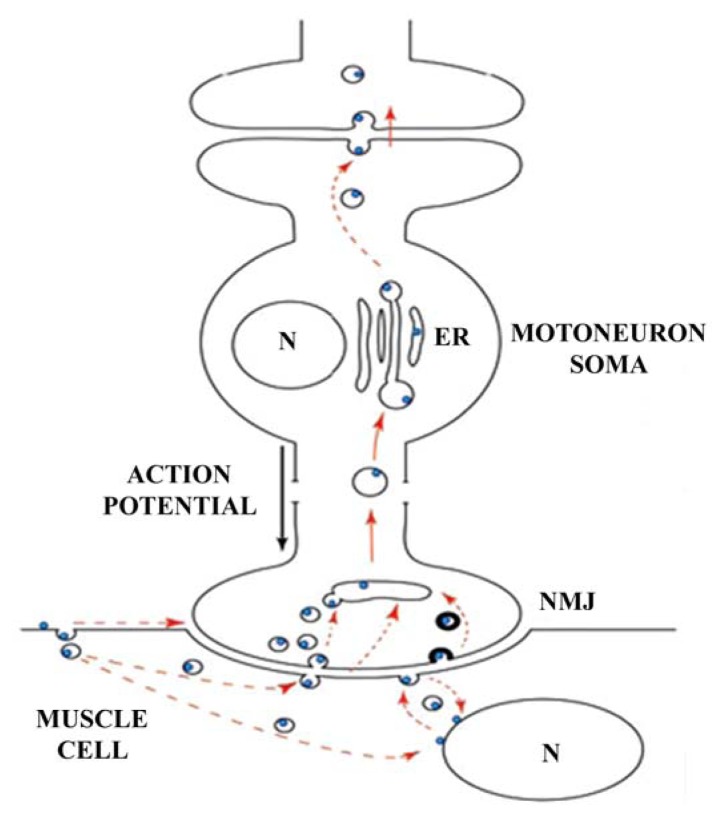
Proposed pathway for the hybrid protein β-Gal-fragment C. Once the hybrid protein is injected intramuscularly, it is found in large uncoated vesicles and then transported retrogradely to the endoplasmic reticulum (ER). Hypothetical pathways are indicated with dashed arrows. Adapted from [63].

One of the most well-known hybrid proteins that has been used for this purpose is the hybrid protein encoded by *lacZ*-fragment C. This protein has been tested *in vitro* and *in vivo* to determine its activity in the hypoglossal system, and the detection of the labeled motor neurons was dependent on time post-injection [60–62]. Since neuronal integrity is crucial for fragment C internalization, the transneuronal molecular pathway at neuromuscular junctions was intensively studied using this hybrid protein [63]. The protein was detected not only in the neuromuscular junction postsynaptic side but also the soma of the motor neuron, away from the active zones in large uncoated vesicles (Figure 6). Other hybrid molecules form multicomponent proteins by recombining fragment C, the translocation domain of diphtheria toxin, and the DNA-binding fragment of the GAL4 transcription factor. This system was particularly effective in PC12 cells [64].

The advances in the understanding of these hybrid proteins have paved the way for new therapeutic approaches using fragment C as a carrier of neurotrophic factors to ameliorate the disease process of motor neuron diseases, neuropathies and pain. Moreover, fragment C represents a potential non-viral vector for delivering exogenous biomolecules, such as proteins and DNA, to neurons *in vitro* and *in vivo* [60]. Among the wide range of neurotrophic factors, such as nerve growth factor (NGF), neurotrophin-3, insulin-like growth factors and vascular epithelial growth factor, the factors most used to construct hybrid proteins with fragment C are brain-derived neurotrophic factor (BDNF) and glial-derived neurotrophic factor (GDNF). More recently, a novel multi-component nanoparticle system using polyethylene imine (PEI) has been evaluated to elicit the expression of BDNF in neuronal cell lines [65].

Additionally, BDNF-fragment C and GDNF-fragment C hybrid proteins exert neuroprotective effects *in vitro* and *in vivo*. For example, GDNF-fragment C promotes neuronal survival and neurite outgrowth in animal models of Parkinson’s disease [66] and amyotrophic lateral sclerosis (ALS) [67,68], and BDNF-fragment C is protective in a mouse model of ALS, although no synergistic effect of this recombinant molecule was found [69].

Similarly, the hybrid protein that contains cardiotrophin-1 and fragment C promoted motor neuron survival *in vitro* in a dose-dependent manner [70], and the combination of the anti-apoptotic molecule Bcl-X_L_ and fragment C improved cell survival and decreased apoptosis in the glutamate-mediated excitotoxicity of SH-SY5Y neuronal cells [71].

Furthermore, fragment C of tetanus toxin and NGF share the same retrograde transport organelles, suggesting neurotrophin receptor p75^NTR^ as the first membrane marker of the retrograde endocytic pathway used by fragment C of tetanus toxin [72]. p75^NTR^ is a transmembrane receptor for neurotrophic factors of the neurotrophin family, which comprises NGF, BDNF and neurotrophin-3 and -4/5. In addition to p75^NTR^, neurotrophins signal, via the tropomyosin-related kinase (Trk) family of receptors, tyrosine kinases Trk A, B and C. The two receptor systems can function synergistically, antagonistically or independently of each other in different cell types [73]. The retrograde pathway of fragment C of tetanus toxin is shared by p75^NTR^, TrkB and BDNF, which is strongly dependent on the activities of the small GTPases Rab5 and Rab7 [56], which suggest that at least a portion of p75^NTR^ is transported toward the soma without undergoing proteolytic cleavage. In particular, an impairment of Rab7 activity inhibits the trafficking of fragment C-, p75^NTR^- and TrkB-containing carriers. However, Rab7 impairment in PC12 cells led to an increase in TrkA and extracellular-regulated kinases (ERK-1/2) phosphorylation and the stimulation of NGF [56].

Considering the potential use of fragment C as a non-viral vector and its likely sharing a common molecular pathway as the one described for the neurotrophic factor BDNF [74], there is speculation about its possible neuroprotective properties. Does fragment C have neuroprotective properties?

## 3. Possible Signaling Pathways for Fragment C of Tetanus Toxin

The molecular pathway involving the activation of the Trk receptors is closely shared by neurotrophic factors and fragment C. The neurotrophin family has been shown to regulate survival, development and functional aspects of neurons in the central and peripheral nervous systems through the activation of one or more of the three members of the receptor tyrosine kinases (TrkA, TrkB, and TrkC) in cooperation with p75^NTR^ [75,76]. NGF can bind to the TrkA receptor or a complex of TrkA and p75^NTR^ [75], BDNF and neurotrophin-4/5 can bind to TrkB, and neurotrophin-3 binds to TrkC. Furthermore, similarly to the specialized internalization of fragment C in a clathrin-dependent process, Schiavo and co-workers proposed that NGF can trigger the recruitment of a pool of p75^NTR^ to clathrin-coated pits to be delivered into the soma of motor neurons, which highlights the regulation of p75^NTR^ signaling in response to neurotrophins [30,77]. In addition, p75^NTR^ can also interact with pro-neurotrophins and, therefore, it serves as a signaling component of the receptor complex for growth-inhibitory molecules of CNS, such as Nogo, prompting Nogo receptor-mediated signaling [78,79].

Aguilera and co-workers described an increase in serotonin synthesis in the central nervous system induced by tetanus toxin, suggesting that the toxin-affected serotonergic innervation in the perinatal rat brain triggered the translocation of calcium-phosphatidylserine-dependent protein kinase C (PKC) [80]. In particular, they found that tetanus toxin, but not botulinum toxin (BoNT/A), produced a specific time- and dose-dependent inhibition of serotonin uptake in rat central nervous system synaptosomes. In fact, tetanus toxin altered a component involving inositol phospholipid hydrolysis, which is associated with PKC activity translocation [81,82]. In addition to this translocation, an enhancement of the tyrosine phosphorylation of the tyrosine receptor TrkA, phospholipase C (PLCγ-1) and ERK-1/2 was also observed [83]. Because fragment C stimulated the PLC-mediated hydrolysis of phosphoinositides in rat brain neurons, this fragment appeared to modulate some signaling pathways involving the transport of serotonin [84].

Furthermore, the activation of intracellular pathways related to the PLCγ-1 phosphorylation and activation of PKC isoforms and the kinases Akt (at Ser 473 and Thr 308) and ERK-1/2 (at Thr 202/Tyr 204) was induced by fragment C in rat brain synaptosomes and cultured cortical neurons. This signal pathway activation was dependent on time and concentration, suggesting that fragment C could exert neuroprotective effects, activating TrkA and TrkB receptors in a similar manner as do NGF and BDNF or neurotrophin-4/5 [85,86].

**Figure 7:**
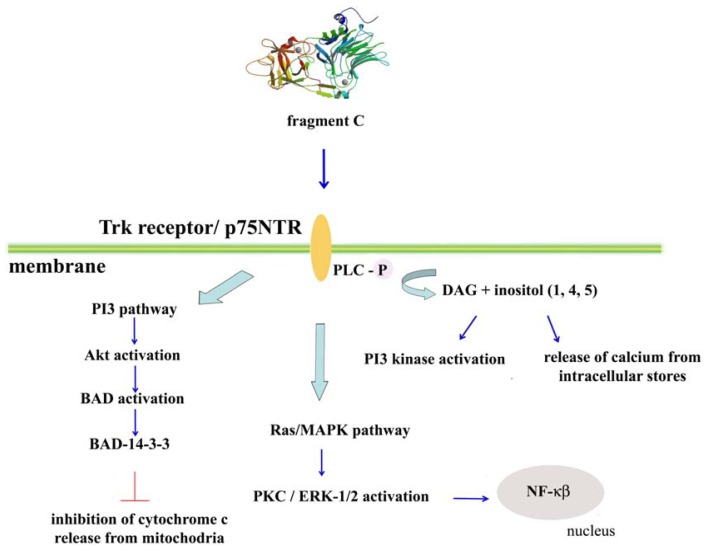
Proposed signaling pathway for fragment C. Based on the results obtained by Aguilera and co-workers, fragment C can induce cerebellar granular cell survival under stress conditions, activating signaling pathways associated with Trk receptors that include the activation of PLC, the Ras/MAPK pathway and the PI3 pathway, leading to the survival of the cell [88,89]. Another possible retrograde pathway of fragment C of tetanus toxin is shared by p75^NTR^, TrkB and BDNF, which is strongly dependent on the activities of the small GTPases Rab5 and Rab7 [56].

Fragment C also protected cerebellar granular cells against potassium deprivation-induced apoptotic death [87] and acted as a neuroprotector in a model of 1-methyl-4-phenylpyridinium (MPP^+^)-triggered apoptosis, enhancing the survival pathways in rats with a dopaminergic lesion and improving different motor behaviors. In this study, fragment C induced Ser 112 and Ser 136 BAD phosphorylation, activated the transcription factor NF-κB, which prevents neuronal death, and induced a decrease in the release of cytochrome c and, consequently, a reduction in the activation of procaspase-3 and chromatin condensation [88,89] (Figure 7).

Consequently, the main advantage of using fragment C as a potential therapeutic agent in non-viral gene therapy is particularly interesting in amyotrophic lateral sclerosis (ALS). The failure of standard treatments in ALS could rely on the inappropriate route of administration and/or the poor bioavailability of molecules to the target cell [90]. The subcutaneous and intrathecal delivery of neurotrophic factors can cause adverse side effects such as weight loss, fever, cough, fatigue and behavioral changes [91], whereas viral gene therapy based on the use of an adeno-associated virus or lentivirus vectors is more efficient than the neurotrophic factor delivery but can induce several inherent hazards [92]. An alternative strategy that effectively reaches motor neurons, can exert neuroprotective properties and does not show such adverse side effects implies the use of fragment C. Osta and co-workers found in a mouse model of ALS, which carries the mutation G93A in human superoxide dismutase 1 (SOD1), transgenic SOD1^G93A^ mice, an amelioration of the decline in hind-limb muscle innervation in the animals that were injected with either naked DNA-encoding fragment C (TTC) or naked DNA, encoding the recombinant molecule fragment C and BDNF (BDNF-TTC). In addition, a significant delay in the onset of symptoms and functional deficits, an improvement in the spinal motor neuron survival (down-regulation of caspase-1 and caspase-3 levels and a significant phosphorylation of serine/threonine protein kinase Akt) and a prolonged lifespan under both treatments was observed [69,93]. Although no significant differences were found between TTC and BDNF-TTC treatments, recombinant plasmid BDNF-TTC was detected in skeletal muscle and the corresponding recombinant protein reached the spinal cord tissue of transgenic SOD1^G93A^ mice, reinforcing on the one hand the carrier properties of fragment C. The active state of BDNF in the recombinant molecule could suggest that BDNF could exert an autocrine and neuroprotective role together with TTC to a similar extent as TTC alone; however this effect could not be sufficient enough to prompt a synergistic effect. As a consequence, BDNF-TTC molecule could mainly use the same pathway that mimics a neurotrophic secretion route, prompting survival signals in the spinal cord of transgenic SOD1^G93A^ mice [66].

Despite all these contributions to the understanding of the signaling pathway of fragment C, further studies should be performed to elucidate its neuroprotective effect.

## 4. Conclusions

Fragment C is considered to be a useful and valuable tool to carry therapeutic molecules due to its efficient retroaxonal transport. This is the main reason why this particular property has been exploited as a therapeutic strategy in the central nervous system to ameliorate the disease process of well-known neurodegenerative diseases, such as Parkinson’s and ALS. However, recent *in vitro* and *in vivo* studies have shown that fragment C can enhance cell survival by itself. This new property of fragment C has opened the door to the understanding of its possible, although not yet well-characterized, molecular pathways. Because the characterization of the protein-protein interaction at the molecular level is of high importance and the plasma membrane of each nervous cell contains a particular composition, a messenger, such as fragment C, can exert different effects depending on the signaling pathway it modulates, which is directly related to the type and distribution of different receptors in the plasma membrane. Thus, a messenger is not the molecule that carries the message; many results can be obtained when studying the same effector molecule under different experimental conditions.

Regarding fragment C, new studies will reveal the precise molecular mechanism by which it can induce its neuroprotective properties. This mechanism could shed light on using fragment C as an alternative therapeutic strategy for more neurodegenerative diseases in the near future.

## References

[1] Johnson J.L., Francis G. (1975). Taxonomy of the clostridia: Ribosomal ribonucleic acid homologies among the species. J. Gen. Microbiol.

[2] Johnson E.A. (1999). Clostridial toxins as therapeutic agents: Benefits of natures’s most toxic proteins. Annu. Rev. Microbiol.

[3] Anderson J.F., Leake J.P. (1915). A method of producing tetanus toxin. J. Med. Res.

[4] Noguchi H. (1907). The nature of the antitetanic action of eosin. J. Exp. Med.

[5] Flexner S., Noguchi H. (1906). The effect of eosin upon tetanus toxin and upon tetanus in rats and guinea-pigs. J. Exp. Med.

[6] Cowie D.M., Greenthal R.M. (1922). Studies on the nature of the action of non-specific protein in disease processes. III. Non-specific proteins and soluble toxin (diphtheria-tetanus). J. Med. Res.

[7] Sherrington C.S. (1905). On reciprocal innervation of antagonistic muscles. VIIIth note. Proc. R. Soc. B.

[8] Acheson G.H., Oscar M.D., Ratnoff D., Schoenbach E.B. (1942). The localized action on the spinal cord of intramuscularly injected tetanus toxin. J. Exp. Med.

[9] Brooks V.B., Curtis D.R., Eccles J.C. (1957). The action of tetanus toxin on the inhibition of motor neurons. J. Physiol.

[10] Firor W.M., Lamont A. (1938). The apparent alteration of tetanus toxin within the spinal cord of dogs. Ann. Surg.

[11] Martini E., Torda C., Zironi A. (1939). The effect of tetanus toxin on the choline esterase activity of the muscles of rats. J. Physiol.

[12] Harvey A.M. (1939). The peripheral action of tetanus toxin. J. Physiol.

[13] Manwaring W.H. (1943). Types of tetanus toxin. Cal. West. Med.

[14] Ipsen J. (1951). The effect of environmental temperature on the reaction of mice to tetanus toxin. J. Immunol.

[15] Wright E.A. (1953). The effect of the injection of tetanus toxin into the central nervous system of rabbits. J. Immunol.

[16] Roaf M.D., Sherrington C.S. (1906). Experiments in examination of the locked jaw induced by tetanus toxin. J. Physiol.

[17] Wassermann A., Takaki T. (1898). Über Tetanusantitoxische Eigenschaften des normalen Centralnervensystems. Berl. Klin. Wochenschr.

[18] Landsteiner K., Botteri A. (1906). Über Verbindungen von Tetanustoxin mit Lipoiden IV. Zbl. Bakt. Orig.

[19] Van Heyningen W.E. (1959). The fixation of tetanus toxin by nervous tissue. J. Gen. Microbiol.

[20] Van Heyningen W.E. (1959). Chemical assay of the tetanus toxin receptor in nervous tissue. J. Gen. Microbiol.

[21] Van Heyningen W.E., Miller P.A. (1961). The fixation of tetanus toxin by ganglioside. J. Gen. Microbiol.

[22] Van Heyningen W.E. (1976). Binding of ganglioside by the chains of tetanus toxin. FEBS Lett.

[23] Sugiyama H. (1980). Clostridium botulinum neurotoxin. Microbiol. Rev.

[24] Johnson E.A. (1999). Clostridial toxins as therapeutic agents: Benefits of nature’s most toxic proteins. Ann. Rev. Microbiol.

[25] Pellizari R., Rossetto O., Schiavo G., Montecucco C. (1999). Tetanus and botulinum neurotoxins: Mechanism of action and therapeutic uses. Philos. Trans. R. Soc. Lond. B.

[26] Montal M. (2010). Botulinum neurotoxin. Annu. Rev. Biochem.

[27] Habermann E., Dreyer F. (1986). Clostridial neurotoxins: Handling and action at the cellular and molecular level. Curr. Top. Microbiol. Immunol.

[28] Chen S., Karalewitz A.P.A., Barbieri J.T. (2012). Insights into the different catalytic activities of *Clostridium* neurotoxins. Biochemistry.

[29] Neubauer V., Helting T.B. (1981). Structure of tetanus toxin: The arrangement of papain digestion products within the heavy chain-light chain framework of extracellular toxin. Biochim. Biophys. Acta.

[30] Deinhardt K., Berninghausen O., Willison H.J., Hopkins C.R., Schiavo G. (2006). Tetanus toxin is internalized by a sequential clathrin-dependent mechanism initiated within lipid microdomains and independent of epsin (eosin?) 1. J. Cell Biol.

[31] Mochida S. (2000). Protein-protein interactions in neurotransmitter release. Neurosci. Res.

[32] Humeau Y., Doussau F., Grant N.J., Poulain B. (2000). How botulinum and tetanus neurotoxins block neurotransmitter release. Biochimie.

[33] Ungar D., Hughson F.M. (2003). SNARE protein structure and function. Annu. Rev. Cell Dev. Biol.

[34] Boquet P., Duflot E., Hauttecoeur B. (1984). Low pH induces a hydrophobic domain in the tetanus toxin molecule. Eur. J. Biochem.

[35] Simpson L.L., Hoch D.H. (1985). Neuropharmacological characterization of fragment B from tetanus toxin. J. Pharmacol. Exp. Ther.

[36] Menestrina G., Forti S., Gambale F. (1989). Interaction of tetanus toxin with lipid vesicles. Effects of pH, surface charge and transmembrane potential on the kinetics of channel formation. Biophys. J.

[37] Calappi E., Masserini M., Schiavo G., Montecucco C., Tettamanti G. (1992). Lipid interaction of tetanus toxin. A calorimetric and fluorescence spectroscopy study. FEBS.

[38] Habermann E., Albus U. (1986). Interaction between tetanus toxin and rabbit kidney: A comparison with rat brain preparations. J. Neurochem.

[39] Lazarovici P., Yanai P., Llavín E. (1987). Molecular interactions between micellar polysialogangliosides and affinity-purified tetanotoxins in aqueous solution. J. Biol. Chem.

[40] Emsley P., Fotinou C., Black I., Fairweather N.F., Charles I.G., Watts C., Hewitt E., Isaacs N.W. (2000). The structures of the H_C_ fragment of tetanus toxin with carbohydrate subunit complexes provide insight into ganglioside binding. J. Biol. Chem.

[41] Sinha K., Box M., Lalli G., Schiavo G., Schneider H., Groves M., Siligardi G., Fairweather N. (2000). Analysis of mutants of tetanus toxin H_C_ fragment: Ganglioside binding, cell binding and retrograde axonal transport properties. Mol. Microbiol.

[42] Louch H.A., Buczko E.S., Woody M.A., Venable R.M., Vann W.F. (2002). Identification of a binding site for ganglioside on the receptor binding domain of tetanus toxin. Biochemistry.

[43] Conway P.M.C., Whittal R.M., Baldwin M.A., Burlingame A.L., Balhorn R. (2006). Electrospray mass spectrometry of NeuAc oligomers associated with the C fragment of the tetanus toxin. J. Am. Soc. Mass Spectrom.

[44] Siade A.L., Schoeniger J.S., Sasaki D.Y., Yip C.M. (2006). *In situ* canning probe microscopy studies of tetanus toxin-membrane interacions. Biophys. J.

[45] Helting T.B., Zwisler O., Wiegandt H. (1977). Structure of tetanus toxin. II. Toxin binding to ganglioside. J. Biol. Chem.

[46] Sutton J.M., Chow-Worn O., Spaven L., Silman N.J., Hallis B., Shone C.C. (2001). Tyrosyne-1290 of tetanus neurotoxin plays a key role in its binding to gangliosides and functional binding to neurons. FEBS Lett.

[47] The 1.61 angstrom structure of the tetanus toxin ganglioside binding region: Solved by MAD and Mir phase combination. www.pdb.org.

[48] Rummel A., Bade S., Alves J., Bigalke H., Binz T. (2003). Two carbohydrate binding sites in the H_CC_-domain of tetanus neurotoxin are required for toxicity. J. Mol. Biol.

[49] Jayaraman S., Eswaramoorthy S., Kumaran D., Swaminathan S. (2005). Common binding site for disialyllactose and tri-peptide in C-fragment of tetanus neurotoxin. Proteins Struct. Funct. Bioinform.

[50] Cosman M., Lightstone F.C., Krishnan V.V., Zeller L., Prieto M.C., Roe D.C., Balhorn R. (2002). Identification of novel small molecules that bind to two different sites on the surface of tetanus toxin C fragment. Chem. Res. Toxicol.

[51] Fotinou C., Emsley P., Black I., Ando H., Ishida H., Kiso M., Sinha K.A., Fairweather N.F., Isaacs W. (2001). The crystal structure of the tetanus-toxin HC fragment complexed with a synthetic GT1b analogue suggests cross-linking between ganglioside receptors and the toxin. J. Biol. Chem.

[52] Jayaraman S., Swaramoorthy S., Kumaran D., Swaminathan S. (2005). Common binging site for disialyllactose and tri-peptide in C-fragment of tetanus neurotoxin. Proteins.

[53] Chen C., Fu Z., Kim J.-J.P., Barbieri J.T., Baldwin M.R. (2009). Gangliosides as high affinity receptors for tetanus neurotoxin. J. Biol. Chem.

[54] Herreros J., Lalli G., Montecucco C., Schiavo G. (2000). Tetanus toxin fragment C binds to a protein present in neuronal cell lines and motorneurons. J. Neurochem.

[55] Herreros J., Ng T., Schiavo G. (2001). Lipid rafts act as specialized domains for tetanus toxin binding and internalization into neurons. Mol. Biol. Cell.

[56] Deinhardt K., Salinas K., Verastigui C., Watson R., Worth D., Hanrahan S., Bucci C., Schiavo G. (2006). Rab5 and Rab7 control endocytic sorting along the axonal retrograde transport pathway. Neuron.

[57] Evinger C., Erichsen J.T. (1986). Transsynaptic retrograde transport of fragment C of tetanus toxin demonstrated by immunohistochemical localization. Brain Res.

[58] Manning K.A., Erichsen J.T., Evinger C. (1990). Retrograde transneuronal transport properties of fragment C of tetanus toxin. Neuroscience.

[59] Fishman P.S., Carrigan D.R. (1987). Retrograde transneuronal transfer of the fragment C of tetanus toxin. Brain Res.

[60] Coen L., Osta R., Maury M., Brûlet P. (1997). Construction of hybrid proteins that migrate retrogradely and transynaptically into the central nervous system. Proc. Natl. Acad. Sci. USA.

[61] Miana-Mena F.J., Muñoz M.J., Ciriza J., Soria J., Brûlet P., Zaragoza P., Osta R. (2003). Fragment C tetanus toxin: A putative activity-dependent neuroanatomical tracer. Acta Neurobiol. Exp.

[62] Miana-Mena F.J., Muñoz M.J., Roux S., Ciriza J., Zaragoza P., Brûlet P., Osta R. (2004). A non-viral vector for targeting gene therapy to motoneurons in the CNS. Neurodegener. Dis.

[63] Miana-Mena F.J., Roux S., Benichou J.C., Osta R., Brûlet P. (2002). Neuronal activity-dependent membrane traffic at the neuromuscular junction. Proc. Natl. Acad. Sci. USA.

[64] Barati S., Chegini F., Hurtado P., Rush R.A. (2002). Hybrid tetanus toxin C fragment-diphtheria toxin translocation domain allows specific gene transfer into PC12 cells. Exp. Neurol.

[65] Oliveira H., Fernandez R., Pires L.R., Martins M.C.L., Simões S., Barbosa M.A., Pêgo A.P. (2010). Targeted gene delivery into peripheral sensorial neurons mediated by self-assembled vectors composed of poly (ethylene imine) and tetanus toxin fragment C. J. Control Release.

[66] Larsen K.E., Benn S.C., Ay I., Chian R.J., Celia S.A., Remington M.P., Bejarano M., Liu M., Ross J., Carmillo P. (2006). A glial cell line-derived neurotrophic factor (GDNF): Tetanus toxin fragment C protein conjugate improves delivery of GDNF to spinal cord motor neurons in mice. Brain Res.

[67] Ciriza J., Moreno-Igoa M., Calvo A.C., Yagüe G., Palacio J., Miana-Mena F.J., Muñoz M.J., Zaragoza P., Brûlet P., Osta R. (2008). A genetic fusion GDNF-C fragment of tetanus toxin prolongs survival in a symptomatic mouse ALS model. Restor. Neurol. Neurosci.

[68] Moreno-Igoa M., Calvo A.C., Ciriza J., Muñoz M.J., Zaragoza P., Osta R. (2012). Non-viral gene delivery of the GDNF, either alone or fused to the C-fragment of tetanus toxin protein, prolongs survival in a mouse ALS model. Restor. Neurol. Neurosci.

[69] Calvo A.C., Moreno-Igoa M., Mancuso R., Manzano R., Oliván S., Munoz M.J., Penas C., Zaragoza P., Navarro X., Osta R. (2011). Lack of a synergistic effect of a non-viral ALS gene therapy based on BDNF and a TTC fusion molecule. Orphanet J. Rare Dis.

[70] Bordet T., Castelnau-Ptakhine L., Fauchereau F., Friocourt G., Kahn A., Haase G. (2001). Neuronal targeting of cardiotrophin-1 by coupling with tetanus toxin C fragment. Mol. Cell. Neurosci.

[71] Carlton E., Teng Q., Federici T., Yang J., Riley J., Boulis N.M. (2008). Fusion of the tetanus toxin C fragment binding domain and Bcl-X_L_ for protection of peripheral-nerve neurons. Neurosurgery.

[72] Lalli G., Schiavo G. (2002). Analysis of retrograde transport in motor neurons reveals common endocytic carriers for tetanus toxin and neurotrophin receptor p75^NTR^. J. Cell Biol.

[73] Ibánez C.F., Simi A. (2012). p75 neurotrophin receptor signaling in nervous system injury and degeneration: Paradox and opportunity. Trends Neurosci.

[74] Roux S., Saint Cloment C., Curie T., Girard E., Mena F.J., Barbier J., Osta R., Molgó J., Brûlet P. (2006). Brain-derived neurotrophic factor facilitates *in vivo* internalization of tetanus neurotoxin C-terminal fragment fusion proteins in mature mouse motor nerve terminals. Eur. J. Neurosci.

[75] Skeldal S., Matusica D., Nykjaer A., Coulson E.J. (2011). Proteolytic processing of the p75 neurotrophin receptor: A prerequisite for signalling? Neuronal life, growth and death signalling are crucially regulated by intra-membrane proteolysis and trafficking of p75(NTR). Bioessays.

[76] Skaper S.D. (2008). The biology of neurotrophins, signalling pathways, and functional peptide mimetics of neurotrophins and their receptors. CNS Neurol. Disord. Drug Targets.

[77] Deinhardt K., Reversi A., Berninghausen O., Hopkins C.R., Schiavo G. (2007). Neurotrophins redirect p75^NTR^ from a clathrin-independent to a clathrin-dependent endocytic pathway coupled to axonal transport. Traffic.

[78] Wang K.C., Kim J.A., Sivasankaran R., Segal R., He Z. (2002). p75 interacts with the Nogo receptor as a co-receptor for Nogo, MAG and OMgp. Nature.

[79] Twiss J.L., Chang J.H., Schanen N.C. (2006). Pathophysiological mechanisms for actions of the neurotrophins. Brain Pathol.

[80] Aguilera J., Lopez L.A., Yavin E. (1990). Tetanus toxin-induced protein kinase C activation and elevated serotonin levels in the perinatal rat brain. FEBS.

[81] Gil C., Ruiz-Meana M., Álava M., Yavin E., Aguilera J. (1998). Tetanus toxin enhances protein kinase C activity translocation and increases polyphosphoinositide hydrolysis in rat cerebral cortex preparations. J. Neurochem.

[82] Inserte J., Najib A., Pelliccioni P., Gil C., Aguilera J. (1999). Inhibition by tetanus toxin of sodium-dependent, high-affinity [^3^H]5-hydroxitryptamine uptake in rat synaptosomes. Biochem. Pharmacol.

[83] Gil C., Chaib I., Pelliccioni P., Aguilera J. (2000). Activation of signal transduction pathways involving TrkA, PLCγ-1, PKC isoforms and ERK-1/2 by tetanus toxin. FEBS Lett.

[84] Pelliccioni P., Gil C., Najib A., Sarri E., Picatoste F., Aguilera J. (2001). Tetanus toxin modulates serotonin transport in rat-brain neuronal cultures. J. Mol. Neurosci.

[85] Gil C., Chaib-Oukadour I., Blasi J., Aguilera J. (2001). H_C_ fragment (*C*-terminal portion of the heavy chain) of tetanus toxin activates protein kinase C isoforms and phosphoproteins involved in signal transduction. Biochem. J.

[86] Gil C., Chaib-Oukadour I., Aguilera J. (2003). *C*-terminal fragment of tetanus toxin heavy chain activates Akt and MEK/ERK signalling pathways in a Trk receptor-dependent manner in cultured cortical neurons. Biochem. J.

[87] Chaib-Oukadour I., Gil C., Aguilera J. (2004). The *C*-terminal domain of heavy chain of tetanus toxin rescues cerebellar granule neurons from apoptotic death: Involvement of phosphatidylinositol 3-kinase and mitogen-activated protein kinase pathways. J. Neurochem.

[88] Mendieta L., Venegas B., Moreno N., Patricio A., Martínez I., Aguilera J., Limón I.D. (2009). The carboxyl-terminal domain of the heavy chain of tetanus toxin prevents dopaminergic degeneration and improves motor behaviour in rats with striatal MPP^+^-lesions. Neurosci. Res.

[89] Chaib-Oukadour I., Gil C., Rodríguez-Álvarez J., Ortega A., Aguilera J. (2009). Tetanus toxin H_C_ fragment reduces neuronal MPP^+^ toxicity. Mol. Cell. Neurosci.

[90] Thorne R.G., Frey W.H. (2001). Delivery of neurotrophic factors to the central nervous system: Pharmacokinetic considerations. Clin. Pharmacokinet.

[91] Borasio G.D., Robberecht W., Leigh P.N., Emile J., Guiloff R.J., Jerusalem F., Silani V., Vos P.E., Wokke J.H., Dobbins T. (1998). A placebo-controlled trial of insulin-like growth factor-I in amyotrophic lateral sclerosis. European ALS/IGF-I Study Group. Neurology.

[92] Check E. (2003). Harmful potential of viral vectors fuels doubts over gene therapy. Nature.

[93] Moreno-Igoa M., Calvo A.C., Penas C., Manzano R., Oliván S., Muñoz M.J., Mancuso R., Zaragoza P., Aguilera J., Navarro X. (2010). Fragment C of tetanus toxin, more than a carrier. Novel perspectives in non-viral ALS gene therapy. J. Mol. Med.

